# Dispensary level pilot implementation of rapid diagnostic tests: an evaluation of RDT acceptance and usage by providers and patients – Tanzania, 2005

**DOI:** 10.1186/1475-2875-7-239

**Published:** 2008-11-19

**Authors:** Holly Ann Williams, Louise Causer, Emmy Metta, Aggrey Malila, Terrence O'Reilly, Salim Abdulla, S Patrick Kachur, Peter B Bloland

**Affiliations:** 1 International Emergency and Refugee Health Branch, Centers for Disease Control and Prevention (CDC), Mail Stop F-60, 1600 Clifton Road NE, Atlanta, GA 30333, USA; 2 National Centre in HIV Epidemiology and Clinical Research, University of New South Wales, Australia; 3 Ifakara Health Research and Development Centre (IHRDC), Tanzania; 4 Malaria Branch, CDC, USA

## Abstract

**Background:**

Malaria rapid diagnostic tests (RDTs) may assist in diagnosis, improve prescribing practices and reduce potential drug resistance development. Without understanding operational issues or acceptance and usage by providers and patients, the costs of these tests may not be justified.

**Objectives:**

To evaluate the impact of RDTs on prescribing behaviours, assess prescribers' and patients' perceptions, and identify operational issues during implementation.

**Methods:**

Baseline data were collected at six Tanzanian public dispensaries. RDTs were implemented for eight weeks and data collected on frequency of RDT use, results, malaria diagnoses and the prescription of antimalarials. Patients referred for RDTs completed a standardised exit interview. Qualitative methods assessed attitudes toward and satisfaction with RDTs, perceptions about the test and operational issues related to implementation.

**Results:**

Of 595 patients at baseline, 200 (33%) were diagnosed clinically with malaria but had a negative RDT. Among the 2519 RDTs performed during implementation, 289 (11.5%) had a negative result and antimalarials prescribed. The proportion of "over-prescriptions" at baseline was 54.8% (198/365). At weeks four and eight this decreased to 16.1% (27/168) and 16.4% (42/256) respectively.

A total of 355 patient or parent/caregiver and 21 prescriber individual interviews and 12 focus group discussions (FGDs) were conducted. Patients, caregivers and providers trusted RDT results, agreed that use of RDTs was feasible at dispensary level, and perceived that RDTs improved clinical diagnosis. Negative concerns included community suspicion and fear that RDTs were HIV tests, the need for additional supervision in interpreting the results, and increased work loads without added compensation.

**Conclusion:**

Overprescriptions decreased over the study period. There was a high degree of patient/caregiver and provider acceptance of and satisfaction with RDTs. Implementation should include community education, sufficient levels of training and supervision and consideration of the need for additional staff.

## Background

Clinical diagnosis of malaria is sensitive but not specific. Over-diagnosis and subsequent over-treatment of patients as a result of clinical diagnosis can lead to increased drug pressure that may facilitate the development of drug resistance. This may also increase costs, particularly with the shift from inexpensive antimalarials (such as chloroquine [CQ] and sulphadoxine-pyrimethamine [SP]) to newer, more expensive drugs. It also exposes patients to the unnecessary risk of adverse events and, among some patients, leaves the real cause of illness untreated. In the current environment of the widespread development of antimalarial drug resistance and limited treatment choices, most endemic countries have changed their antimalarial drug policies to newer artemisinin-based combination therapies. Because of the increased cost of these combination therapies and the need to use them wisely in order to prolong their usefulness, improvement of malaria diagnosis has become a critical consideration when formulating rational malaria drug polices.

Having access to a test that quickly confirms the presence of malaria parasites could enable the health care provider to determine whether prescription of antimalarials is appropriate. Microscopy, though still considered the gold standard for malaria diagnosis, is not available at most health facilities. Quality control measures and supervision are often inadequate or absent, staff are poorly trained, and equipment is missing or is in need of repair. Furthermore, clinicians have been known to distrust microscopy results [1-4].

Malaria rapid diagnostic tests (RDTs) show promise as a diagnostic tool that requires minimal training and equipment and provides rapid results. RDTs use immunochromatographic methods to detect antigens derived from malaria parasites in lysed blood. In controlled settings, RDTs have generally been reported to achieve sensitivities and specificities of > 90% in the detection of *Plasmodium falciparum *at densities above 100 parasites/μL blood. Below this level, sensitivity decreases markedly. Among the advantages of the RDT are their ease of use and interpretation. They do not require any electricity or special equipment. It has been demonstrated in the field that simplified, brief training can result in good retention of skills and minimal inter-user variability[5,6]. Furthermore, test kits can be shipped and stored at ambient conditions, although exposure to high temperatures (as seen in most malaria endemic areas) can cause the RDT to degrade. Recommended storage temperatures by the manufacturers tend to fall in the range between 4°C and 30°C but few health care facilities in resource-poor settings have air-conditioning or temperature controlled storage units for drugs or supplies[7]. Findings from a study that examined temperatures during both transport and storage of RDTs in Cambodia and the Philippines showed that transport and storage temperatures both greatly exceeded the recommended temperatures for field use[7]. In spite of these challenges, in many situations where microscopy services are not adequately maintained (in terms of both equipment and personnel), the accuracy of RDTs is certain to be superior to clinical diagnosis. Despite their relatively high unit cost ($0.60 – 2.50)[5], RDTs to diagnose malaria are now being viewed as an important component of rational malaria case management.

If used appropriately, RDTs could improve clinical prescribing practices and reduce the potential for the development of drug resistance. RDTs are beginning to be implemented in clinical settings but the reality is that many end-users will be using RDTs in rural clinics with minimal supervision. Little is known about the acceptance and usage of RDT results by providers and patients/caregivers or field operational issues related to the implementation of RDTs in the periphery. In a recent study, Reyburn and colleagues demonstrated that more than 90% of prescriptions for antimalarial drugs in a low-moderate transmission setting were for patients for whom the malaria test was negative[2]. Their findings also indicated that use of a RDT, along with training in the use of the test, did not in itself lead to a lower level of over-treatment for malaria. RDTs can only promote rational drug use if providers are willing to use the findings to guide their prescribing behaviors and if patients and parents/caregivers accept the subsequent treatment (particularly if antimalarials are not prescribed). Preliminary findings from recent RDT studies in Kenya, Tanzania and Zambia indicated that 35.5 – 54% of patients with a negative RDT received antimalarials[2,3] (personal communication, J. Skarbinski, CDC, 2007). This reinforces other studies whose findings have documented that up to 79% of patients with negative blood smears were prescribed antimalarials [1-4]. Without understanding whether these tests can and will be accepted and used appropriately, the cost of the tests may not be justified and cost-savings from more rationale use of antimalarials may not be realized.

Socio-behavioural studies have previously documented that one factor that influences a provider's behavior is community and/or individual pressure[8,9]. Providers will often prescribe drugs that have been specifically requested by patients or caregivers. Alternatively, many rural health facilities have a very limited number of alternative drugs and providers may simply prescribe antimalarials as nothing else is available. Thus, if antimalarials are prescribed despite a negative RDT result, it is important to identify the factors (social or otherwise) that weighed more heavily on the prescriptive behaviors than the test results. Understanding provider attitudes and behaviors may assist in anticipating the impact of RDTs on malaria case management and help guide training and implementation of these tests.

Although RDTs are simple to use and provide rapid results, there are operational issues that should be considered. Factors that may influence RDT results include such things as buffer and blood volume, age and storage of test kits and blood samples, visual acuity of person conducting and interpreting the test, levels of parasitemia, and levels of temperature and humidity [10-12]. Trained staff are required to perform and interpret test results, yet in many situations, including much of sub-Saharan Africa, RDTs will be placed in facilities where staff are poorly educated, staff attrition may be high and little supervision exists on a routine basis. Rural dispensaries in Tanzania are often staffed by one or two persons who need to evaluate 40 or 50 patients a day and the introduction of a test that requires even an additional 15–20 minutes to perform may be considered burdensome. It will be important to understand the strengths and weaknesses of RDTs under such circumstances, how best to incorporate RDTs into the routine management of patients at such facilities, and who is best suited to perform the RDT. In districts where IMCI (Integrated Management of Childhood Illness) is implemented (i.e. a formal system of clinical diagnosis where a child with a history of fever anytime within the previous 48 hours, feels hot, or temperature > 37.5°C is considered to have malaria), introduction of an RDT is likely to face additional operational issues. During a technical visit to assess implementation of RDTs and ACTs in nine health facilities in the rural Rufiji district of Tanzania, it was recently noted that, in spite of having IMCI implemented, health care providers continued to prescribe RDTs for children under the age of five years, rather than following the IMCI guidelines. Investigating the impact of RDTs in these different settings is important to identify operational issues that may impact the implementation of RDTs[13].

Lastly, little is known about patients/caregivers' perceptions of treatment options offered based on the results of RDTs. In the best scenario, patients/caregivers would perceive the tests as an important diagnostic service that would assist the prescriber in more accurately determining the cause of their or their child's illness. This in turn might improve patient adherence to antimalarials (in particular the newer combinations that often have more complex dosing regimens). However, for many years, patients in Africa received antimalarials for any source of fever. Thus, expectations for receiving medications, particularly antimalarials, are high and patients/caregivers often express their desire for specific drugs. Unless they perceive the RDTs as a positive improvement in their health care, they may be reluctant to accept the proposed treatments that are based on the RDT results, particularly if the test is negative and they have fever. If this happens, they may seek antimalarials from private sources, such as pharmacies, private clinics or drug stores.

Tanzania has recently implemented artimisinin-based combination therapy (ACT), specifically using artemether-lumefantrine (Coartem^®^), for first-line treatment of uncomplicated malaria. To strengthen malaria diagnosis and promote rational drug use, an assessment was done to evaluate the impact of RDTs on prescribing behaviors and malaria case management, assess prescribers' and patients' perceptions about the use of RDTs, and identify operational issues that impact the use of RDTs at dispensary level in Tanzania.

## Methods

### Study site and sample

The study was conducted in 2005 in Mkuranga District (a rural coastal district within Pwani Region, located approximately 30 kilometres south of the capital city, Dar es Salaam), an area of year round transmission, with seasonal peaks in May and June. Data from the 2004 Demographic and Health Survey[14] indicated that 40.3% children under five years of age in Pwani Region reported fever within 14 days of the survey. Of these, 71 reported using an antimalarial drug within 48 hours of fever. IMCI was in use in this district at the time of the pilot implementation. The pilot implementation and subsequent evaluations coincided with the onset of the high transmission season in this district.

Of a possible 14, a convenience sample of six rural, public dispensaries were purposively chosen with the help of the Council Health Management Team (CHMT) based on: a) geographical range and logistic feasibility in reaching the areas during rainy season, b) having adequate facility utilization rates in order to achieve the desired sample size and c) representation of the five "cascade" areas (areas of CHMT supervision). These facilities represented the lowest level of peripheral care – none had microscopy services at the time of the study.

### Study Implementation, data collection and analysis

Both quantitative and qualitative methods were used to evaluate this pilot implementation of RDTs over an eight week period.

#### Baseline data collection

Following training, data were collected sequentially on all patients attending the selected dispensaries, regardless of diagnosis, as part of a baseline health facility survey (HFS). The following were excluded: woman known to be pregnant, trauma patients, those presenting with signs and symptoms of severe malaria, and those refusing or unable to provide informed consent or assent. A sample of approximately 100 patients per dispensary (total n = 600) was estimated to be required for evaluation RDT quality, using an expected sensitivity of 99% and a least acceptable sensitivity of 95% with 95% confidence and 80% power. This sample size calculation was based upon an expectation that approximately 34% of patients with a clinical diagnosis of malaria would be parasitemic and RDT positive[15]. This sample size was also sufficient to evaluate other health facility survey variables.

Following the consultation with the provider, a study team member conducted an exit interview with eligible patients that collected information on demographics, presenting symptoms, diagnoses and treatments prescribed. Following this exit interview, a study laboratory technician collected a finger-prick blood sample, prepared a thick and thin blood film for malaria microscopy and performed a RDT. Results were recorded on a standardized case reporting form. The patient register, maintained by the dispensary staff, was reviewed by study staff to gather information on the number of patients seen per day, diagnoses recorded and treatments prescribed.

#### Microscopy

Microscopy was considered the gold standard. Staining and microscopy was conducted by a study microscopist stationed at one of the study sites. Microscopic examination of the blood was done according to standard techniques. Slides were stained with 10% Giemsa and leukocytes were counted in the same fields until 200 leukocytes were counted. Parasite densities were calculated using an assumed leukocyte count of 6,000 leukocytes per μl of blood.

A second expert microscopist, stationed at the district hospital, reviewed blood films where there were discordant microscopy and RDT results. If there was further discordance between the two study microscopists, a third study microscopist stationed at a different district in the same region, reviewed the film. All microscopists were blinded to the results of the corresponding RDT and the readings of the other microscopists.

#### Malaria RDTs

"Paracheck Pf," manufactured by Orchid Biomedical Systems (India), was chosen with input from the Tanzanian NMCP and because it complied with WHO quality control standards. This test used a histidine-rich protein-2 (HRP-2) detection system for *P. falciparum*, with reported sensitivities of 99% and specificities of 100% for *Plasmodium falciparum *by the manufacturer. The Malaria Laboratory Research and Development Unit at the Centers for Disease Control and Prevention tested sensitivity and specificity against known samples of varying density and heat stability (as recommended by WHO), using a selection of RDTs from the same lot as used in the implementation and found them to meet the manufacturer's stated sensitivity and specificity. The results of the test are read 15 minutes after the test has been conducted. This RDT was used during the baseline HFS according to the manufacturer's instructions at the selected dispensaries.

#### RDT training

Following the HFS and prior to RDT implementation at the selected sites, RDT training was conducted with the dispensary staff that would be involved with using the RDTs in this evaluation over a two-day period and included information about diagnosis and treatment algorithms, RDT referral criteria, and RDT use (including general information, limitations of test, how to perform the test, test result interpretation, troubleshooting, storage and handling of the test). It also included a session focused on practice using the RDT on known positive and negative samples. The level of training provided exceeded what has generally been provided at other sites of implementation, which often has been quite minimal or not described well in implementation studies[2,3,16-18].

Facility staff were responsible for all aspects of the RDT implementation and each dispensary identified the staff person that would be conducting and interpreting the RDT results. The training for the providers was developed with input from the Mkuranga District Council Management Health Team in order to mimic local conditions of how a new diagnostic technique would be introduced. Algorithms based on the Tanzanian Malaria Treatment Guidelines were used as training guides for best practice but, as in actual practice, during the duration of the study, the providers made independent decisions about clinical management of each patient. During training, providers were instructed that a positive RDT result indicated malaria and were encouraged to prescribe antimalarials as per national guidelines. With a negative RDT result, providers were instructed that this indicated no malaria and other causes of febrile illnesses should be investigated. In this case, no antimalarials should be prescribed but other treatments should be utilized as indicated. Providers were encouraged to consider RDTs for all eligible patients (at three dispensaries – all ages, at the other three dispensaries – only those over five years of age) with a suspected clinical diagnosis of uncomplicated malaria for RDTs during the subsequent eight weeks.

#### RDT implementation

Upon completion of training, sufficient supplies of RDTs (Paracheck) were distributed to each dispensary. Use of RDTs was at the discretion of the facility staff. Although study staff visited each dispensary during implementation to observe the process, document operational concerns related to implementation, and during evaluation periods obtain consent and collect daily study recording forms, there was minimal interactive supervision from the study staff. So as to approximate normal working conditions, the study team did not supply watches or clocks to the dispensaries; thus, each person conducting the test devised a way to monitor or estimate the time required for reading of the test.

#### Daily data collection

Throughout the eight weeks of implementation, dispensary staff maintained daily registers recording, by individual patient, whether a RDT was performed, the RDT results, diagnosis made, and the treatment prescribed. Data from these registers were collected and compiled by study staff and summarized daily onto standardized forms. Variables, by day, included age category of patient (≤ or > 5 years of age), number of RDTs performed, RDT results, and antimalarials prescribed.

#### Analysis of quantitative data

Data were entered in Tanzania at Ifakara Health Research and Development Centre using Microsoft Fox Pro software for quantitative data and Microsoft Word for qualitative data (Microsoft, Seattle, WA, USA). Quantitative data were analysed using SAS V8 (SAS Institute Inc., Cary, NC, USA). Frequency distributions were calculated and differences in proportions were assessed by Chi-square or Fisher's exact tests. RDT results were characterized by true positive, true negative, false positive and false negative as compared to microscopy.

"Correct" prescription of antimalarials is defined as those with positive RDT who were prescribed antimalarial and those with negative RDT who were not prescribed antimalarials. "Overprescription" is defined as those with a clinical diagnosis of malaria and who were RDT negative, but who were prescribed antimalarials.

#### Qualitative evaluation periods

There were two qualitative evaluation periods during implementation. These occurred during weeks four and eight. During these periods, data were only collected on the first 80 patients seen.

#### Qualitative instrument development

All qualitative interview guides were developed with input from the Tanzanian supervisory staff on the project. Guides were translated into KiSwahilli and back translated to English, followed by pilot testing. Modifications to language and procedures were completed as necessary.

#### Qualitative interviews

During the two evaluation weeks (four and eight of the pilot implementation period), 25–30 patients referred for RDTS per dispensary were recruited to participate in qualitative exit interviews. For patients under age five years, parents or caregivers were interviews. Once consented, exit interviews were conducted in KiSwahilli (primary language for Tanzania) and later transcribed to English. All providers who used the RDTs were asked to participate in individual interviews and all facility staff that were involved in any aspect of using the RDTs were asked to participate in informal discussions at each dispensary. Field notes were recorded during these discussions and later transcribed for analysis. The majority of the provider interviews and informal discussions were conducted in KiSwahli. The interviews were transcribed and translated to English by members of the study team. Using content analysis techniques, themes were identified from the interview data. Frequency counts were done on each identified theme to determine the strength of the theme. Data were examined across the six sites and the two evaluation periods by the lead author.

### Human subjects' protection

The research protocol was approved by institutional review boards at CDC and the National Medical Research Coordinating Committee at the National Institute for Medical Research in Tanzania. Informed consent for all procedures and interviews was obtained from all study participants and there were no refusals.

## Results

### Sensitivity and specificity

Among the baseline survey of the population (n = 595), clinical diagnosis, as compared to gold standard microscopy, had a sensitivity of 74.5% and specificity of 45%. RDTs had a sensitivity of 94.4% and specificity of 88.6% compared to microscopy for infections with parasite density greater than 100 parasites per uL.

### Baseline health facility survey

595 patients attending the selected six dispensaries in Mkuranga District were surveyed. Selected characteristics of the surveyed population are presented in Table 1. Of these, 196 (32.9%) were under five years of age. Overall clinical malaria (as determined by the provider) was reported in 365 (61.3%) of the surveyed population. Parasite prevalence (as determined by microscopy) was 31.9% (95% confidence interval [CI] 28.2–35.7) with a geometric mean parasite density of 2076 parasites per uL.

**Table 1:** Characteristics of survey population (n = 595)

**Characteristic**	**n (%) [95% CI]**
< 5 years of age	196 (32.9)
Clinical malaria	365 (61.3)
Parasite prevalence	190 (31.9) [28.2–35.7]
Geometric mean parasite density	2076 [1563.5–2757.0]

Of the 595 patients included in the baseline HFS, 165 (28%) with a clinical diagnosis of malaria had a positive RDT, 200 (33%) with a clinical diagnosis of malaria had a negative RDT, 172 (29%) did not have a clinical diagnosis of malaria and had a corresponding negative RDT, while 58 (10%) with no malaria diagnosis had a positive RDT.

Among those with a clinical diagnosis of malaria (n = 365), 200 (55%) had a negative RDT, representing the proportion of "overdiagnosis" in this survey population. Overdiagnosis was observed in 44 (30%) and 156 (72%) of participants aged < 5 (n = 196) and ≥ 5 (n = 399) years of age respectively.

Among those prescribed antimalarials (in this survey, all those with a clinical diagnosis of malaria, n = 356), sulphadoxine-pyrimethamine was the most commonly prescribed (70%), followed by amodiaquine (27%), and quinine (1%). No ACTs or chloroquine were prescribed.

### Implementation of RDTs

Each facility determined the staff member who would be responsible for implementing the RDTs. All six facilities obtained watches on their own in order to read the test result at the correct time specified by the manufacturer.

### Appropriate use of RDTs by providers

Over the eight week implementation period, 2,519 RDTs were performed. Among those having an RDT performed, 1,329 (52.8%) had a positive test and were prescribed antimalarials and 879 (34.9%) had a negative test and no antimalarials were prescribed; 289 (11.5%) with a negative test had antimalarials prescribed, while 22 (0.9%) had a positive test but no antimalarials prescribed.

**Figure 1:**
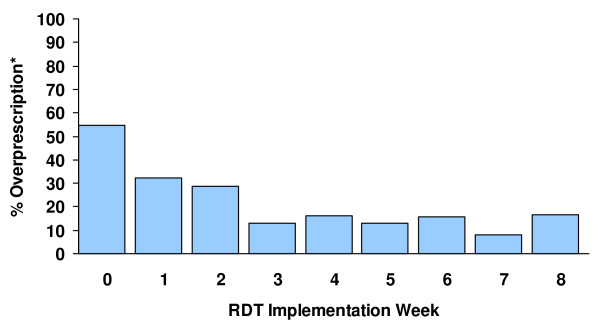
Proportion of antimalarial overprescriptions* by RDT implementation week.

Figure 1 presents the percent of 'over-prescriptions,' defined as the proportion of those with a negative RDT who were prescribed antimalarials among all those prescribed antimalarials, by week of implementation. Prior to implementation the proportion of "over-prescriptions" based upon the baseline HFS was 54.8% (n = 198/365). At week four of implementation, the proportion had decreased to 16.1% (n = 27/168) and at week eight, it was 16.4% (n = 42/256).

**Figure 2:**
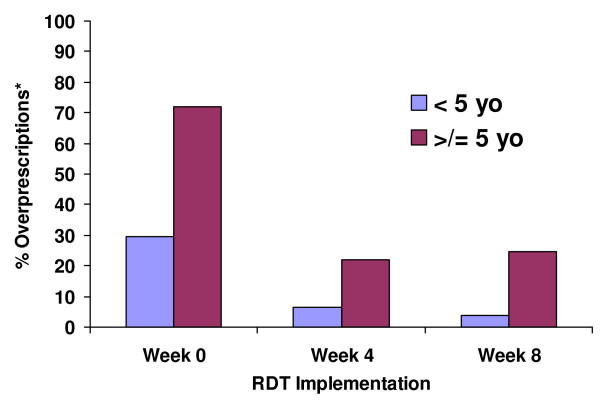
Proportion of overprescriptions by age category at RDT implementation weeks 0, 4 and 8.

Figure 2 shows the proportion of over-prescriptions by age at baseline, week four, and week eight of implementation. Over-prescription was substantially higher among those equal to and above five years of age; however, the proportion declined across both age categories over the eight week period of the study, from 29.5% to 3.9% among those < five years of age and from 71.9% to 24.7% among those ≥ five years of age.

### Perceptions of RDTs by providers

#### Individual provider interviews

Twenty-one providers were interviewed (ten during week four, 11 during week eight), most of them clinical officers with an average of eight years of clinical experience. There was remarkable similarity in findings across sites and evaluation periods.

The majority of the providers (n = 16, 76.2%) had had no previous experience with any type of rapid diagnostic test. Virtually all providers agreed that RDTs improved practice, were feasible for use at dispensary level and enhanced appropriate treatment. The providers perceived that patients were happy with the test, as the patients felt that RDTs improved treatment. The test was seen as being quick, easy to use and simple. The majority of providers agreed that the tests made their jobs easier as they were now more confident with their diagnoses.

There was unanimous agreement from the providers that they trusted the results, however, this did not mean that the test results were used in all situations to guide prescribing behaviors. Although agreeing that they trusted the test results, two clinical officers noted that they relied on clinical symptoms, rather than test results, to guide prescribing practices. Their opinions did not change over the course of the eight week period of implementation.

A key theme from the providers was that diagnosis was easier as the providers could focus on identifying alternate reasons why a patient might be febrile if the RDT result was negative. One provider noted that, by week three, he focused on other potential diagnoses as he saw how many patients were returning with negative test results. As one provider explained:

"A patient describes his problems....and you may have more than one impression. Malaria and urinary track infections (UTI) have similar symptoms. If you make a test and find that the patient does not have malaria, you can then think of UTI and treat it. This simplifies my treatment decisions."

#### Informal discussions with facility staff

Results from the informal discussions with the facility staff echoed the findings from the provider interviews. The tests were viewed in a positive light as they were perceived to improve the process of diagnosis and patient satisfaction was high. Staff noted that improved patient satisfaction resulted in large numbers of patients coming to the clinic and better utilization of their facility. Staff also discussed that news about the RDTs had spread rapidly throughout neighbouring villages and patients who were not ill with fever came to the dispensaries asking to be tested for malaria. However, staff were quick to point out that, in spite of people wanting the test who were not febrile, the providers were appropriately using the tests only for 'eligible' patients (as defined by the study guidelines).

In both weeks four and eight, staff noted the continued fears of some members of the communities that the RDTs were, in actual practice, tests for HIV/AIDs, although they clarified that these concerns lessened over time. While staff indicated that the tests were good in that the provider could feel confident making a malaria diagnosis, they also said that use of antimalarials for RDT negative patients continued to occur.

#### Operational issues

Data from the interviews, informal discussions and field notes from the study staff indicated that some operational issues needed attention during the implementation period. For example, sometimes watches used to time the results did not function properly. During one non-evaluation week, one dispensary's watch stopped operating properly, so staff either asked patients to time the results or they guessed when to read the test result, which resulted in reading the test results prematurely (observations by the field staff at the site).

Staff commented that they felt it took a few weeks of practice to really 'feel comfortable' with using and interpreting the RDTs. They repeatedly mentioned the issue of the test results changing over time, i.e., if they re-examined the test after the allotted 15 minute interval, they would get a different result. The issue of changing test results after 15 minutes was discussed at both points of evaluation. One clinical officer summarized, in his quote below, the feelings of many of the providers regarding the problem of a change from a 'negative' to a 'positive' test:

*"You wait to read the result until it is 15 minutes and you see nothing *[negative] *but after sometime when you read it again, you find it is positive with a very thin bar on the testing window. You prescribe accordingly, when it is negative you give aspirin and the patient leaves but then you read it after awhile and you find it has changed into positive. What are we to do? Maybe this is for patients with scant malaria."*

Some staff mentioned that they had difficulty collecting the appropriate volume of blood – too much blood applied to the test cassette subsequently obscured the test strip, making the test result unreadable. They also felt that the tests were difficult to read in ambient light, particularly during heavy rains. One recurrent problem that facility staff encountered while using the RDTs, was reading the cassette when the result line was faint or 'thin,' particularly if the person doing the reading had poor eyesight. Junior staff needed to consult with senior staff, even up to the eighth week of implementation, on interpreting the test results that they felt were ambiguous (the results in question were weak positives). With practice, these concerns lessened.

One village health worker described how he dealt with the issue of results that were difficult to read (see quote below). The strategy of adding additional minutes prior to reading the test was a common strategy used in these dispensaries:

*"Some RDTs, when you wait 15 minutes, they don't give you results until you wait for 20 up to 30 minutes...unless you do extra work of looking/checking *[checking the test thoroughly and properly]. *I learned some things, for instance, instead of using 15 minutes, I used to add one, two or four minutes until I get accurate results."*

Providers also discussed the problem of RDT-negative patients going to different facilities that had microscopes, where they were told that they had malaria. The providers openly questioned whether they had misdiagnosed a patient due to a faulty RDT or whether the issue was in faulty microscopy readings. Compounding this problem were the patients who insisted on being given antimalarials, even if they had tested negative. As one clinical officer described:

*"I had a patient who had symptoms of malaria and I ordered him to be checked first. After the test results were negative, I told him it could be due to tiredness and so he should use paracetamol first. He refused and begged me to give him SP *[sulphadoxine-pyrimethamine]. *He said that the test lies...if we have people that do not understand, then they force us to comply with their demands...they insist to be given antimalarials."*

Most staff perceived that the test put a strain on normal operations and additional staff were needed. At one dispensary, during one day of the first evaluation period, all staff members, except the Clinical Officer (CO), were absent. The CO felt that the shortage of staff made use of RDTs impossible due to the large patient load. All patients during this day were treated clinically. As well, a smaller number of facility staff felt that staff remuneration should also be increased, as there was a perception that health facility utilization rates had climbed during the time of the RDT implementation. Due to the frequent absence of staff for illness or trainings, facility staff learned that, rather than assigning the responsibility of the test to one staff person, it was necessary that responsibility for the RDTs be rotated among staff, including down to the level of nurse's aides and, in one dispensary, the village health volunteer.

### Perceptions of RDTs by patients/caregivers

As with the providers, there were marked similarities in the data, during both evaluation periods and across all six sites. A total of 355 patients/caregivers were interviewed (175 week four, 180 week eight). Of these, 59.2% (210) were women. Of those interviewed, 90% (n = 317, 3 missing data) had received a diagnosis of clinical malaria, with fifty-three percent (n = 186, 1 missing) confirmed with a positive RDT result.

When asked what they thought of the RDT, most participants were quite positive about the test. Patients/caregivers commented that the test allowed the provider to identify the disease [literally meaning identifies whether malaria is present or not]; thus, it allowed the provider to better match the treatment with the disease, which decreased the use of unnecessary drugs. The test was also seen as being 'fast.'

The vast majority of participants (97.2%, n = 345) said that they trusted the RDT results. The main reasons given for trust in the results centered on: a) the test was endorsed by experts (providers, laboratorians, outside experts and the Ministry of Health) and b) the test results matched the symptoms experienced by the patients.

However, there was an element of conditional trust in these results. When asked to explain their response about trusting the RDTs, it was clear that the RDT result was trusted if the test results matched signs and symptoms of illness and, thus, confirmed the patients' own view about their illnesses. The trust was also predicated on the prescribed treatment resulting in the patients improving or becoming 'cured.'

Five of the eight who did not trust the results said it was because their symptoms indicated they had malaria so, therefore, the test must be incorrect. Three were indecisive, illustrated by this comment from a patient:

"I feel malaria but the result is negative. Really, I do not know if it is accurate or not. But it seems it is inaccurate. Why should it be negative when I have all the malaria symptoms?"

Patients/caregivers were asked whether they would be wiling to use or have the test used, as an additional proxy question reflecting trust. The vast majority (n = 345, 97.2%) of patients/caregivers agreed that they would use the RDTs again. The major reasons cited were that the test confirms whether malaria is present or not, thus allowing for use of the most appropriate drugs. They also noted that the test should be given before taking any medications. As one person commented:

"When you get a test, you will be sure with the problem facing you and which treatment will be appropriate to the illness, rather than using drugs without any test."

For those who would not take the test (n = 5), the main reason given was that microscopy was better because you could actually get a count of the parasites.

### Patient/caregiver concerns

Initially, there were marked fears that the test would be used to identify HIV/AIDS and not malaria. Staff needed to reassure patients that this was a malaria rapid test prior to getting patients and caregivers to agree to use the RDT.

### Recommendations by providers and patients/caregivers

All participants in the study were asked for their ideas on improving RDT implementation. Facility staff recommended hiring additional staff and providing more training and supervision during RDT implementation. Technical recommendations included improving the test so that it reflected all species and parasite density, as well as designing it so that 'weak positive' results were not so problematic for test result interpretation. Providers encouraged scaling-up RDT implementation so that all facilities could have access to this diagnostic tool.

More than a third (n = 132, 37.2%) of the patient/caregivers commented that RDTs should be implemented programmatically at all levels of health care facilities. In addition, patients/caregivers felt that RDTs should be provided for free. Lastly, they mentioned their hope for development of additional rapid tests to help diagnosis other illnesses.

## Discussion

RDTs remain an exciting new intervention for malaria control. Clinical diagnosis leads to over-diagnosis of malaria and subsequent over-prescription with antimalarials, particularly among older children and adults as confirmed in this study and others[2,3,10,19,20]. This study also demonstrated that over-diagnosis and -prescription may be reduced through the use of RDTs among this population at peripheral dispensaries in rural Tanzania. Though not representative, these results are encouraging and warrant further RDT implementation, supported by appropriate monitoring and evaluation of this technology.

Findings from the baseline HFS (prior to RDT implementation) conducted in the selected dispensaries indicated that 55% of those with clinical malaria had no evidence of malaria parasitaemia by RDT. This population also represented the proportion being "over-treated" and, thus, were the potential target for reduction through the use of RDTs. Over-treatment decreased from 55% to 16.1% during the first four weeks, with this lower level of overtreatment appearing to be sustained over the following weeks of implementation. This decrease in "overtreatment" over time may reflect providers increasing confidence with RDT use and their results, an explanation supported by providers' comments during the qualitative assessments of RDT implementation. A small proportion of patients (n = 22, 0.9%) who were RDT-positive did not receive an antimalarial. This occurred in spite of pre-implementation training, which included a suggested treatment algorithm that was approved by the NMCP. By age group, the reductions in overtreatment were similarly encouraging. Patients ≥ five years of age appeared to be the group with the greatest proportion of overtreatment at baseline (~72%) compared to those < 5 years of age (~30%). Following eight weeks of implementation, the proportion of overtreatment for those > five years of age had decreased to ~25%, and among < 5 years of age to ~4%.

A promising finding was the overall positive appraisal of the test by the staff involved with the RDTs, in spite of initial concerns about the ability to correctly read the results. This enthusiasm for the test echoed other recent findings in the literature[18].

Ignoring negative RDT results and consequently treating non-malarial fevers with expensive ACTs is a realistic scenario. In addition to the wasted costs of the RDTs and ACTs, doing so may jeopardize the well-being of patients by ignoring other potentially life-threatening diseases that may be responsible for the febrile episodes. By not providing appropriate treatment, misdiagnosed illness episodes may become more costly in terms of repeat visits to the clinics, use of inappropriate medications by private vendors and loss of time from school or employment[21]. Additional emphasis on non-malaria febrile illness diagnosis and management needs to be given in order to help providers consider alternate reasons for fever so that appropriate non-malaria case management can occur[3].

As well, systems need to be in place for monitoring how often patients with negative RDT results go to different facilities where they are given clinical microscopy results that are contradictory to the RDT results. If patients are convinced that they have malaria based on how they feel, they will, most likely, seek alternative ways to confirm their own perceptions. Health education messages have always been that microscopy is the 'gold standard' and, thus, microscopy is often perceived as a higher level of care than what is routinely given at the most peripheral level. However, given the poor overall state of microscopy in most government facilities in peripheral areas, there is a strong chance that smear readings may be inaccurate, resulting in continued unnecessary use of antimalarials. The problem is that patients, caregivers and even most peripheral health care workers do not have the technical skills or expertise to determine the underlying cause of discrepant test results. There is a potential for increased mistrust in RDT results, should a lot of patients seek alternate malaria confirmation using unreliable microscopy results. To prevent or lessen this possibility, Ministries of Health need to improve facility-based microscopy, in conjunction with RDT implementation[22]. Expertise in microscopy is also essential for providing quality control for the introduction and maintenance of RDTs, providing parasite counts and diagnosing other diseases[23,24].

Ill patients want to have a diagnosis for their illnesses. The RDT allowed patients and caregivers to quickly learn whether malaria was the cause or not, which allowed them to narrow the possibilities of what might be causing their symptoms. Satisfaction with having a rapid diagnosis was also seen in other RDT implementation studies[16]. However, community social pressure for antimalarials is a factor that needs to be considered when training providers not to prescribe antimalarials with a negative test result. In time, as RDT-negative individuals begin to experience clinical improvement without the use of antimalarials or with treatment for other non-febrile illnesses, this social pressure may wane. Until then, training should include methods, such as role playing, that might help providers to better cope with patient expectations.

It is not clear why some providers noted that they used patients' clinical presentations to guide prescribing practices, rather than the test results, in spite of saying that they trusted the results. This may be due to language translation of the term 'trust' or simply a loss of novelty with a new intervention and reverting to what had been standard practice. For years presumptive diagnosis has been the 'rule' in situations where laboratory diagnostics were not available. Training in the use of RDTs should incorporate attention to the shift in clinical decision-making that will be needed when faced with a patient who is RDT negative but has symptoms compatible with malaria. As well, clinicians will need training to assist their patients in understanding why different clinical management decisions are being made when the patient firmly believes that their symptoms indicate malaria.

The type of training that was provided to the dispensary level staff was, in all likelihood, more extensive than what could be provided during a national roll-out of RDTs. Although it has been suggested that novice technicians can competently use RDTs with minimal training[17,25], it was observed in this study that closer supervision, particularly during the first week, was needed in order to assure the health care workers that their techniques were appropriate. Comments continued up to the eighth week that more training and supervision was needed. For RDT implementation to be successful, implementation planning should include a minimum of two days of training, followed by regular monthly facility supervision (or bi-weekly during the first several weeks of implementation). Routine evaluations should be planned to determine if/how the test results are affecting prescribing behaviors.

In spite of the more intensive training prior to implementation and the presence of study staff on-site, the qualitative data indicated that some staff continued to believe that the 'real' results were seen after the recommendation time period for reading results. Qualitative findings from a recent study that examined operational issues related to use of RDTs in South Africa also confirmed that staff struggled with issues such as not reading the test in a timely manner or using too much blood. As well, it was noted that RDTs were in use in spite of insufficient training for the staff conducting and interpreting the tests[18]. Even with manufacturing instructions translated to a local language and pre-implementation training by a research team in Myanmar, village health volunteers reported having insufficient instructions for test use during a study of RDT implementation[25]. Given the variability in time in which test results were read in this study and others, it is critical that use of the test, as per the manufacturing instructions, be stressed in trainings for use of RDTs.

Additional data are needed regarding the best length of training to offer (particularly on-site) and the type and frequency of supervision. Without attention to these issues pre-implementation, it would be necessary to have fairly detailed monitoring on-site in order to determine if staff were using the test and interpreting the results correctly. Reading the cassette lines was problematic and individuals coped with this by simply increasing the length of time to read the test; thus, potentially increasing the chances that the test result may not be appropriate. Given the personnel and financial constraints that most Ministries of Health operate under, particularly in sub-Saharan Africa, it may be more feasible to roll-out the intervention slowly in areas in which supervision will be difficult to provide. The study findings suggest that a longer period of time than originally thought may be needed so that staff can become comfortable in using the tests, before they are expected to have them as part of their daily service provision. Offering examples of the types of problems that may occur (such as problems timing the test) could be included in the initial training so that the facilities can begin to think about ways to address common problems.

Prior to national implementation, issues that need attention include developing: a) effective distribution and management systems that take into account the need for temperature-controlled distribution and storage, b) appropriate instructions for staff with low levels of literacy, assuming that levels of supervision will be minimal, c) case management strategies for determining RDT eligibility and management of RDT results, and d) quality assurance and control systems, particularly considering the likelihood that the RDTs will be stored in less-than-ideal settings[10,11,26]. As well, given the mistrust of microscopy results as noted earlier, strengthening microscopy diagnostic services should improve clinicians' trust in microscopy and improve the likelihood that clinicians accept diagnosis results[3]. Appropriate ways to incorporate training and supervision for both microscopy and RDTs should be assessed. Likewise, although data from modeling suggests that RDTs have the potential to be cost-effective compared to presumptive treatment[23,27], additional research is needed to evaluate the cost-effectiveness of these tests when used operationally in various settings and over extended time periods.

### Limitations

There are several limitations that should be mentioned. This pilot implementation was quite brief and may be an insufficient period of time to really understand the dynamics surrounding provider behavior in relationship to implementing RDTs. The implementation for this evaluation focused solely on government health facilities, so the results cannot be extrapolated to use of RDTs by private providers. A study staff member was physically on-site at all dispensaries during the entire pilot implementation and all facilities were visited numerous times by the study supervisory staff, which reflects a "best case scenario" and one that, most likely, will not occur outside of a research study environment. This level of assistance is unrealistic when compared to the minimal levels of supervision that can be realistically provided by the district health management teams. The level of training provided as part of this study may have contributed to the positive outcomes observed, however, this was not formally assessed. During the implementation and evaluation periods, there were no stock outs of tests or drugs as RDTs and first-line treatment (SP, not the more expensive ACTs) were always available. Providers were encouraged throughout implementation to complete a patient register, modified from the government issued patient register, which recorded information about patient demographics (age and sex), diagnosis, information about why RDTs were or were not ordered, RDT results and treatment prescribed. The process of completing this log or register may have positively influenced use of RDTs and their results. Even so, these interventions may be necessary to guarantee the appropriate use of RDTs and their results. Finally, this study was conducted at rural dispensaries where microscopic diagnosis and trained laboratory staff were unavailable prior to the introduction of RDTs. It is possible that the culture of doubting frequently inaccurate blood slide results described elsewhere[1,2] was less important a factor in this setting.

## Conclusions and recommendations

Introduction of RDTs should be considered to improve rational drug use of newer, more expensive antimalarial drugs. Prior to full-scale implementation of RDTs, there are important operational issues that should be considered. An expanded training of health care workers should be developed, including the use of clear guidelines to encourage proper use of RDTs and their results. Identification of common potential problems in the field and possible solutions may be of great value. It is vitally important that, particularly in resource-poor countries, the development of training and supervision plans include not only members of the malaria control programmes and policy makers but also front-line laboratorians and health staff that will be using and interpreting the tests. These discussions should realistically examine the context in which these tests will be implemented[22].

The results of this study suggest that RDTs for malaria can be implemented effectively and may be highly acceptable to health care workers, patients and caregivers. The level of training and supervision provided in this evaluation may have been greater than other studies, which showed less promising results[2]. As much as this might limit the generalizability of the study findings, it also underscores that many public health officials and malaria policy makers may have underestimated the intensity of training and supervision required to ensure adequate deployment of RDTs for malaria. On-site supervision during the beginning stages of implementation is critical, particularly focusing on assistance with reinforcing the recommended timing required for reading of the test results and result interpretation. Clinical algorithms, with a focus on the management of other non-malarial febrile illnesses, should be included in the training and reinforced during supervisory visits so that providers can appropriately apply case management. Health utilization rates should be followed closely over time to determine if additional staff are needed. Community health education should be instituted prior to introduction of the RDTs so that patients/caregivers understand what the test is and how the results will be used. Lastly, as there remains relatively little information on the use of RDTs over time, additional operational research should be conducted to examine if use or acceptance of the test alters over time and what measures are necessary to maintain it and assure quality control in real world delivery systems.

## Competing interests

The authors declare that they have no competing interests.

## Authors' contributions

HAW: co-investigator, conception of study, research protocol development, over-all project supervision, data analysis, development and critical review of manuscript.

LC: co-investigator, conception of study, research protocol development, over-all project supervision, data analysis, development and critical review of manuscript.

EM: field supervision and data collection, review of manuscript.

AM: field supervision and data collection.

TO-R: field supervision and data collection.

SA: conception and design of the study, review of manuscript.

SPK: conception and design of the study, review of manuscript.

PBB: conception and design of the study, review of manuscript.
